# Supplementary material for: Fatigue resistant jaw muscles facilitate long-lasting courtship behaviour in the southern alligator lizard (Elgaria multicarinata)
Source: Proc Biol Sci. 2020 Sep 23;287(1935):20201578. doi: 10.1098/rspb.2020.1578 (PMC7542809; doi:10.1098/rspb.2020.1578)
Supplement: Table S1 and Table S2 [file rspb20201578supp1.pdf]

Fatigue resistant jaw muscles facilitate long-lasting courtship behavior in the southern alligator lizard (*Elgaria multicarinata*)

Allyn Nguyen, Jordan P. Balaban, Emanuel Azizi, Robert J. Talmadge, A. Kristopher Lappin

Correspondence to aklappin@cpp.edu

Proceedings of the Royal Society B

DOI: 10.1098/rspb.2020.1578

Table S1. Antibodies used for immunohistochemistry in lizard thigh muscle. Fibers 1, 3, 4, 8 exhibit pattern A slow characteristics. Fibers 2, 5, 6, 7 exhibit pattern B slow characteristics. Fibers 9, 10, 11, 12 exhibit four different patterns of fast characteristics. Positive (+) = reaction; negative (-) = no reaction.

	Antibody										
**Fiber**	**2F4C**	**71**	**A4.15.19**	**ALD 58**	**BF 13**	**D5**	**BF F3**	**N3.36**	**Sigma Fast**	**Vector Slow**	**Pattern**
1	-	-	-	+	-	+	-	-	-	+	A slow
2	-	-	-	+	-	-	-	-	-	-	B slow
3	-	-	-	+	-	+	-	-	-	+	A slow
4	-	-	-	+	-	+	-	-	-	+	A slow
5	-	-	-	+	-	-	-	-	-	-	B slow
6	-	-	-	+	-	-	-	-	-	-	B slow
7	-	-	-	+	-	-	-	-	-	-	B slow
8	-	-	-	+	-	+	-	-	-	+	A slow
9	-	-	+	+	+	-	-	+	+	-	C fast
10	-	-	-	-	-	+	-	+	+	-	D fast
11	-	-	+	-	+	+	-	+	+	-	E fast
12	-	-	+	-	-	-	-	-	+	-	F fast

Table S2. Antibodies used for immunohistochemistry in lizard jaw muscle reacted with Mabs. 2F4c = anti-MHC IIm, A4.15.19 = anti-MHC IIa, ALD-58 = anti-MHC I. Positive (+) = reaction; negative (-) = no reaction.

	Antibody									
**Fiber**	**2F4C**	**71**	**A4.15.19**	**ALD 58**	**BF 13**	**D5**	**BF F3**	**N3.36**	**Sigma Fast**	**Vector Slow**
1	+	-	+	+	-	-	-	-	-	-
2	+	-	+	+	-	-	-	-	-	-
3	+	-	+	+	-	-	-	-	-	-
4	+	-	+	+	-	-	-	-	-	-
5	+	-	+	+	-	-	-	-	-	-
6	+	-	+	+	-	-	-	-	-	-
7	+	-	+	+	-	-	-	-	-	-
8	+	-	+	+	-	-	-	-	-	-
9	+	-	+	+	-	-	-	-	-	-
10	+	-	+	+	-	-	-	-	-	-
11	+	-	+	+	-	-	-	-	-	-
12	+	-	+	+	-	-	-	-	-	-
